# Potential of Single Pulse and Multiplexed Drift-Tube Ion Mobility Spectrometry Coupled to Micropillar Array Column for Proteomics Studies

**DOI:** 10.3390/ijms23147497

**Published:** 2022-07-06

**Authors:** Cindy Nix, Gael Cobraiville, Marie-Jia Gou, Marianne Fillet

**Affiliations:** Laboratory for the Analysis of Medicines (LAM), Department of Pharmacy, CIRM, University of Liege, Avenue Hippocrate 15, B36 Tour 4 +3, 4000 Liège, Belgium; cindy.nix@uliege.be (C.N.); gael.cobraiville@chuliege.be (G.C.); mjgou@uliege.be (M.-J.G.)

**Keywords:** drift-tube ion mobility spectrometry, multiplexing, proteomics, micropillar array column

## Abstract

Proteomics is one of the most significant methodologies to better understand the molecular pathways involved in diseases and to improve their diagnosis, treatment and follow-up. The investigation of the proteome of complex organisms is challenging from an analytical point of view, because of the large number of proteins present in a wide range of concentrations. In this study, nanofluidic chromatography, using a micropillar array column, was coupled to drift-tube ion mobility and time-of-flight mass spectrometry to identify as many proteins as possible in a protein digest standard of HeLa cells. Several chromatographic parameters were optimized. The high interest of drift-tube ion mobility to increase the number of identifications and to separate isobaric coeluting peptides was demonstrated. Multiplexed drift-tube ion mobility spectrometry was also investigated, to increase the sensitivity in proteomics studies. This innovative proteomics platform will be useful for analyzing patient samples to better understand unresolved disorders.

## 1. Introduction

Omics’ approaches aim to study a large number of molecules in order to better understand biological systems. Omics’ technologies target genes (genomics), transcripts (transcriptomics), proteins (proteomics) or metabolites (metabolomics) [1]. Over the past few decades, the interest in biomarker discovery, disease diagnosis, prognosis, personalized medicine and treatment follow-up has grown exponentially [2,3]. The human proteome contains thousands of proteins distributed in a wide range of concentrations and having very different physico-chemical properties [4]. Due to this enormous complexity, it is crucial not only to have powerful mass spectrometer (MS) devices and acquisition methods, but also to maximize peptides’ separation, prior to their entrance into the MS. 

Nowadays, the complexity of the proteome samples is addressed through increasingly resolutive mass analyzers, such as Orbitrap and Time-of-Flight (TOF). Besides, many developments have also been made in terms of MS acquisition. Among them, data-independent acquisition (DIA) constitutes one major advance for proteomics’ analysis. Commonly, bottom-up proteomics data are acquired using data-dependent acquisition (DDA), in which the most abundant ions observed during the survey scan (MS1) are selected for fragmentation, according to their charge state (MS2) [5]. Considering the wide range of proteins’ concentrations in the human proteome, the identifications in DDA are biased toward the most abundant proteins. Regarding DIA, all of the precursor ions are fragmented in MS2, without any preselection. Therefore, DIA constitutes an interesting approach for targeting low abundant proteins [6]. Several types of DIA modes are available nowadays, to perform proteomics studies [7,8]. 

Ion-mobility technologies coupled with mass spectrometry have also brought substantial improvement in the proteomic workflows [9,10]. Among them, trapped ion mobility spectrometry (TIMS), coupled to a TOF analyzer, constitutes one major development of the last ten years. In TIMS, the ions are immobilized in a moving gas, thanks to an electric field gradient (EFG). The position of immobilization into the gas depends on the mobility of each ion. Once enough ions have been immobilized into the TIMS funnel, the EFG is decreased at a defined rate to push the ions out of the TIMS funnel. In TIMS, the ions are separated according to their mobility in a gas phase [11]. This technology can be used with the parallel accumulation–serial fragmentation (PASEF) operation mode [12]. This mode allows a considerable increase in the MS/MS scan rate and therefore the sequencing speed, without a loss of the sensitivity [13]. In the present study, another interesting type of ion-mobility technology was used, namely drift tube ion mobility (DTIMS) [14]. In this configuration, the ions are moving in a stationary gas phase thanks to an electric field, instead of being immobilized into a moving-gas phase, such as in TIMS. By coupling DTIMS to nanofluidic liquid chromatography (nano LC) and TOF analyzer, a third dimension of separation is added, where molecules are separated according to their size, shape and charge in the millisecond range [6,15]. 

Besides these developments in MS acquisition, improvements in separation technologies have also emerged. In 2017, Pharmafluidics (Ghent, Belgium) commercialized innovative octadecylated nano LC columns, called micro pillar array columns (µPAC) [16]. These columns, produced by microlithographic etching on a silicon chip, have a highly ordered backbone composed of pillars [17]. This type of column offers several advantages, such as high permeability, good stability, low batch-to-batch variability and high retention times’ reproducibility [18]. Additionally, the back pressure generated by the passage of the mobile phase through the column is low, which allows to increase considerably the column length (2-m long columns are available) [19]. These innovative nano chromatographic columns drastically increase the number of proteins identified in complex samples, compared to the classical packed nano LC columns, thanks to their higher peak capacity [15,19]. For more exhaustive information about this technology, Rozing recently reviewed the properties and characteristics of the µPAC columns [17]. Since their commercial introduction, these columns have mainly been used for proteomics applications, including recent interest in single-cell proteomics analysis [18]. However, they have also been used in other fields, such as lipidomics or the quality control of biopharmaceuticals [17]. Several applications using µPAC columns in proteomics were developed in the last few months. As an example, Furket et al. used a 2-m long µPAC column to better understand the signaling pathways of inositol phosphates [20]. Kristensen et al. studied the contamination issues of isolation methods used to study liposomal protein corona [21]. Pucci et al. developed piezoelectric nanoparticles to treat glioblastoma multiforme, a highly aggressive brain tumor [22]. Merckaert et al. conducted proteomics analysis in LC–MS, using a 50 cm µPAC column to better understand the role of AKT-2 in breast cancer [23]. Prajapati et al. analyzed bile samples [24] and Hamouda et al. studied the mammalian polyamine transport system [25]. 

## 2. Results and Discussion

In this work, µPAC columns were coupled to DTIMS–QTOF to maximize the number of identified peptides and proteins in complex samples, using DDA and DIA acquisition modes. A number of chromatographic parameters were first optimized. To increase the spray stability, a chip was used as spray emitter after the µPAC column. Then, the potential of DTIMS to separate the coeluting isobaric peptides was evaluated. Finally, for the first time, the interest of the multiplexed DTIMS applied to the analysis of the complex proteome digest was investigated. 

### 2.1. Chromatographic Optimization

Three parameters of the nano LC method were optimized, since they would have an important impact on the number of identified peptides and proteins. First, the comparison of the C18 trapping columns, one made of silica particles, the other one made of micropillars, was performed. Then, the composition of the sample-resuspending solvent, as well as the sample-loading mobile phase, was studied. Finally, the impact of the column length was evaluated by comparing the performances of a 2 m and a 50 cm long column. Other parameters, such as gradient composition and gradient time, were already investigated in a previous study [15].

#### 2.1.1. Comparison of Two Trapping Columns

**Figure 1:**
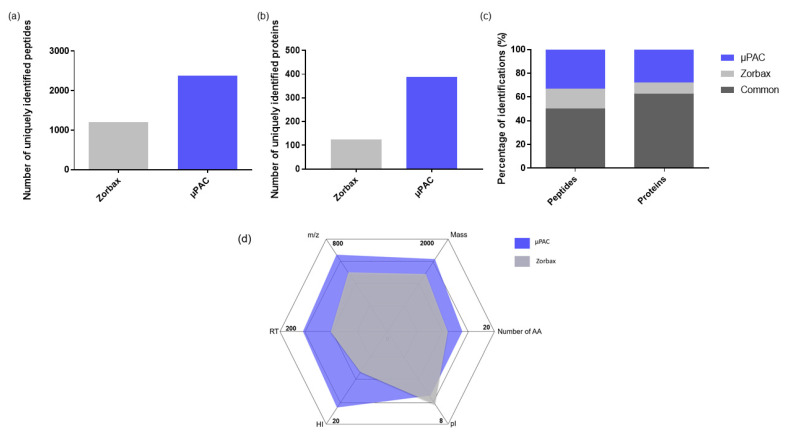
(**a**) Number of peptides uniquely identified with C18 Zorbax trapping column and C18 µPAC trapping column; (**b**) Number of proteins uniquely identified with C18 Zorbax trapping column and C18 µPAC trapping column; (**c**) Percentage of peptides and proteins identified with both trapping columns, uniquely with Zorbax trapping column and uniquely with µPAC trapping column; (**d**) Kiviat diagram of the physicochemical properties of peptides identified only with Zorbax trapping column (grey) and only with µPAC trapping column (blue). The displayed numbers correspond to pool of triplicate injections. Analytical column: 2 m µPAC. Sample: 1 µg Pierce™ HeLa Protein Digest Standard resuspended in H_2_O containing 0.1% FA. Mobile phases of the capillary pump containing 0.1% FA.

Recently, a µPAC trapping column, having the same type of backbone as the µPAC analytical column, was commercialized by Pharmafluidics. In this study, this new µPAC trapping column was compared to a packed-bed Zorbax C18 trapping column, that was used in previous studies for proteomics analysis [15]. As expected, more of the peptides and proteins were identified with the µPAC trapping column (Figure 1a–c). Due to the high internal volume of the µPAC trapping column (1 µL compared to 0.23 µL for Zorbax), more peptides can be trapped, explaining to a large extent the higher number of identifications. Nevertheless, the two trapping columns were found to be complementary. Indeed, the physicochemical properties of the peptides uniquely identified with the Zorbax C18 trapping column and the µPAC trapping column were compared (Figure 1d). As shown in this figure, the peptides that identified uniquely with the µPAC trapping column are longer and have a higher mass, *m*/*z*, retention time (RT) and hydrophobicity index (HI). Besides, their isoelectric point is on average lower than those of the peptides uniquely identified with the Zorbax C18 trapping column. The differences in the physicochemical properties observed between the peptides uniquely identified by the µPAC trapping column or the Zorbax trapping column can be explained by the fact that the µPAC trapping column is end-capped [26], while this is not the case for the Zorbax trapping column [27]. Consequently, the reduced number of free silanols might explain that the µPAC trapping column retains more hydrophobic peptides compared to the Zorbax trapping column. Those results indicate that the use of both the µPAC and Zorbax trapping columns could be interesting to maximize the number of identifications. However, the use of different trapping columns to analyze the same sample would require a high amount of the sample, which is often challenging in proteomics. Besides, performing multiple analyses of the same sample would also be time consuming. For those reasons, µPAC trapping column was selected for the next experiments.

#### 2.1.2. Influence of the Acidic Modifier in Sample Loading Mobile Phase and Sample Resuspending Solvent

**Figure 2:**
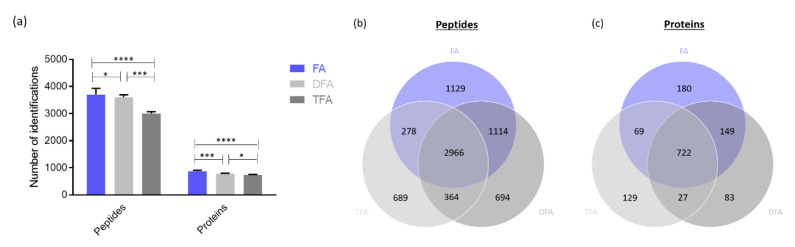
(**a**) Number of peptides and proteins identified with the different acidic modifiers: FA, DFA, TFA. (mean ± SD (*n* = 3)) * represents *p*-value < 0.05; *** (*p*-values < 0.001) and **** (*p*-value < 0.0001); (**b**) Venn diagram representing the number of peptides identified with FA, DFA or TFA in sample-loading mobile phase and the sample resuspending solvent (pool of three runs); (**c**) Venn diagram representing the number of proteins identified with FA, DFA or TFA in the sample-loading mobile phase and the sample resuspending solvent (pool of three runs). Analytical column: 2 m µPAC. Sample: 1 µg Pierce™ HeLa Protein Digest Standard.

The impact of the acidic modifier used in the sample-loading mobile phases and in the sample resuspending solvent was evaluated in terms of peptide–protein identifications. The number of peptides and proteins annotated using 0.1% FA, DFA or TFA were compared using a Tukey’s multiple comparison test. As a result, significantly more peptides and proteins were identified using FA compared to DFA or TFA (Figure 2a). Figure 2b,c show the complementarity of FA, DFA and TFA in terms of identifications. Forty-one percent of the peptides and fifty-three percent of the proteins identified are common to the three acids. 

FA and TFA are the most used mobile phase modifiers in LC–MS. TFA is well known for reducing the signal in electrospray ionization and to produce spray instability [28]. Despite that, TFA enhances the chromatographic performances due to its ability to form pairs of ions. Ion pairing reduces the peak width and increases peak capacity [28,29]. DFA is an alternative mobile phase modifier, which constitutes a compromise between the chromatographic performances and the ion suppression effect. Indeed, DFA has been described as providing ion-pairing properties, combined with good ionization in electrospray [29]. Regarding our results (Figure 2), the signal intensity was reduced with the addition of TFA or DFA in the resuspending solvent and the mobile phases, while no advantage in terms of peptides’ recovery was obtained. Therefore, FA was kept for the next investigations. 

#### 2.1.3. Optimization of the Sample Resuspending Solvent

**Figure 3:**
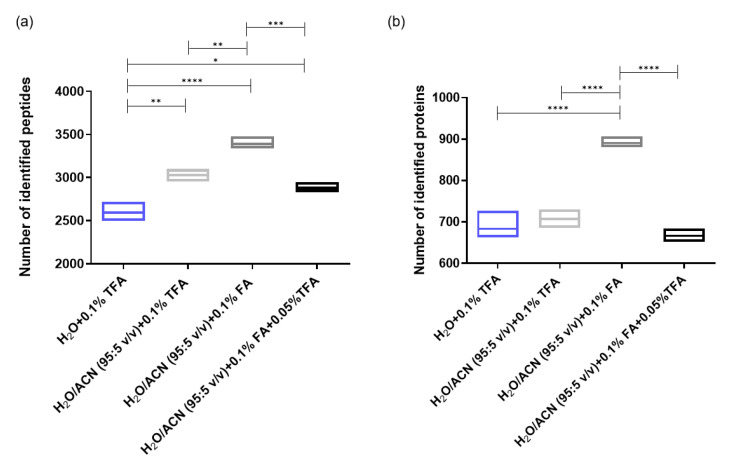
(**a**) Number of peptides identified with different resuspending solvents; (**b**) Number of proteins identified with the different resuspending solvents. Line at mean and boxes from min to max (*n* = 3). * represents *p*-value < 0.05; ** (*p*-value < 0.01); *** (*p*-values < 0.001) and **** (*p*-value < 0.0001). Sample: 1 µg Pierce™ HeLa Protein Digest Standard.

Four different sample resuspending solvents were tested. The number of identified peptides and proteins using the different resuspending solvents were compared, using a Tukey’s multiple comparison test. The resuspending solvent containing H_2_O/ACN/FA (95:5:0.1 *v*/*v*/*v*) allowed the identification of significantly more peptides and proteins than the three other resuspending solvents (Figure 3). Interestingly, the addition of 5% of acetonitrile in the resuspending solvent increased the solubility of most of the hydrophobic peptides and decreased the absorption of these peptides on the surface of polypropylene vials [30]. 

Since TFA is a well-known ion-pairing agent, used to increase the retention of hydrophilic peptides on an RP stationary phase [31,32], an increased number of identifications could therefore be expected. However, a lower number of identifications was obtained using TFA in the resuspending solvent compared to the presence of FA only. The retention of the hydrophilic peptides paired with TFA could be too strong to allow the elution of these peptides from the trapping column. 

#### 2.1.4. Comparison of Two µPAC Column Lengths

**Figure 4:**
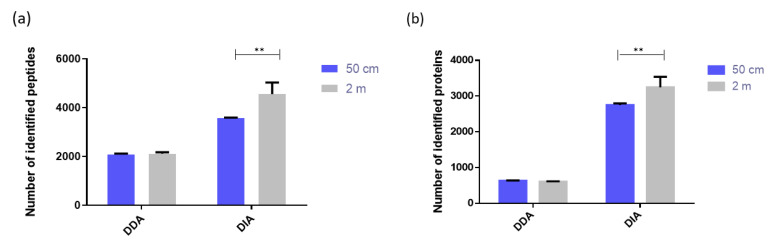
Comparison of the number of identified peptides (**a**) and proteins (**b**) using the 50 cm and 2 m long µPAC columns (*n* = 3). ** (*p*-value < 0.01). Sample: 500 ng Pierce™ HeLa Protein Digest Standard.

In this study, the two µPAC column lengths commercially available, namely 2 m and 50 cm, were compared by analyzing 500 ng of HeLa Protein Digest Standard. The impact of the column length on the number of peptides’ and proteins’ identifications was studied, using DDA and DIA modes. As expected, significantly more peptides and proteins could be identified with the 2 m µPAC column, compared to the 50 cm µPAC column (Figure 4). Nevertheless, the enhancement factor was relatively low. Indeed, the 2 m µPAC column allowed for the identification of 1.18 times more peptides and 1.14 times more proteins, compared to the 50 cm µPAC column. This small increase in identifications was obtained at the cost of a large increase in analysis time (155 min for the 50 cm µPAC column and 310 min for the 2 m µPAC column). For those reasons, the 50 cm long column constitutes a good compromise and was selected for further investigations that aimed to analyze large cohorts of clinical samples.

### 2.2. Interest of Using a Chip as Spray Emitter 

In this study, a chip was used as an emitter, instead of a conventional silica needle typically used in nano LC. This chip is made of polyimide films and contains no packing material for chromatographic separation. It is usually used for direct infusion experiments [33]. In the present study, this chip was connected to the end of the µPAC column thanks to the HPLC–chip cube interface. The performances obtained with a silica needle and the chip spray emitter were compared. In terms of number of peptide and protein identifications, peak height, peak width (FWHM) and signal-to-noise ratio, no statistically significant difference was observed between the two types of emitter. However, in term of ease of use, the chip largely supplants the silica needles (Figure S1, Supplementary Materials). Indeed, the spray obtained with the chip is more stable, so there is less risk of encountering technical problems leading to the loss of precious samples. This aspect is crucial in proteomics studies dealing with a low amount of valuable patient samples. Besides the spray stability, the HPLC–chip cube interface allows an easy connection between the µPAC column and the emitter, limiting the risk of dead volume that can be encountered by connecting the µPAC column to a silica needle. The chip constitutes, therefore, an interesting approach to increase the robustness of nano LC–MS systems. 

### 2.3. Data-Dependent Acquisition and Data-Independent Acquisition Driven by DTIMS

**Figure 5:**
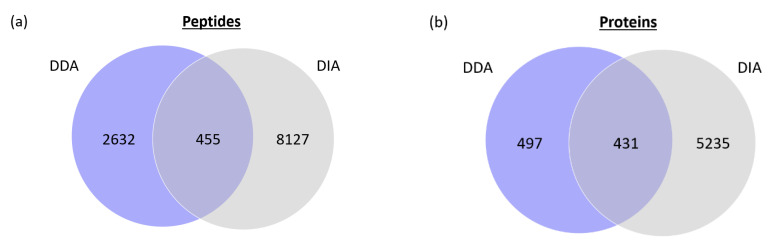
(**a**) Venn diagram representing the number of peptides identified in DDA and DIA; (**b**) Venn diagram representing the number of proteins identified in DDA and DIA. Displayed numbers are pool of peptides and proteins obtained in three runs. Analytical column: µPAC 50 cm; column temperature: 25 °C; Sample: 500 ng Pierce™ HeLa Protein Digest Standard.

Two acquisition modes were used in this study to analyze the human proteome of HeLa cells, namely data-dependent acquisition (DDA) and data-independent acquisition (DIA). As previously observed on *E. coli* digest samples [6], the complementarity between these two acquisition modes in terms of peptides and proteins identifications was demonstrated (Figure 5). DIA provides many more identifications compared to DDA, but the use of DDA is still relevant since it provides unique information. The relatively low overlap between the peptides and proteins identified in DDA and DIA could be explained by the high complexity of the sample. Indeed, even using DIA and the most powerful mass spectrometers, all of the peptides and proteins present in such a complex sample cannot be identified. The higher number of identifications obtained in DIA could be explained by the fact that there is no precursor selection, based on the abundance in DIA, while that is the case in DDA [34]. Interestingly, drift-tube-based DIA displayed a high potential to increase the proteome coverage, especially in complex samples, such as human proteome digests. However, the peptide identification is not straightforward in the DIA mode, compared to DDA [6]. Instead of using one software (Spectrum Mill) to identify the peptides and proteins, it requires a specific and time-consuming workflow (Figure S2, Supplementary Materials) involving several types of software to finally obtain a list of the identified peptides and proteins. Another contribution from DIA is the ability to provide the CCS values of the peptides, which can be incorporated into a DDA-based library. This type of homemade library usually contains the sequence of the peptides, their mass and their retention time. The addition of their corresponding CCS value can increase the confidence level of peptide annotation in complex samples [35,36]. In this study, the obtained CCS values were satisfying in terms of repeatability. Indeed, the CCS value of 500 peptides were compared across three runs and the coefficient of variation obtained was lower than 0.29%. 

### 2.4. Interest of Drift-Tube Data-Independent Acquisition in Proteomics

In proteomics, the separation of the isobaric and co-eluting peptides remains an important challenge [37,38]. Indeed, these peptides have the same retention time and *m*/*z* ratio. They might have different amino acid sequences, or be isomers containing the same amino acid sequence but a different D/L enantiomeric ratio. Several studies have shown that peptides comprising R amino acids could play a key role in the development of age-related diseases, among others [37]. 

**Figure 6:**
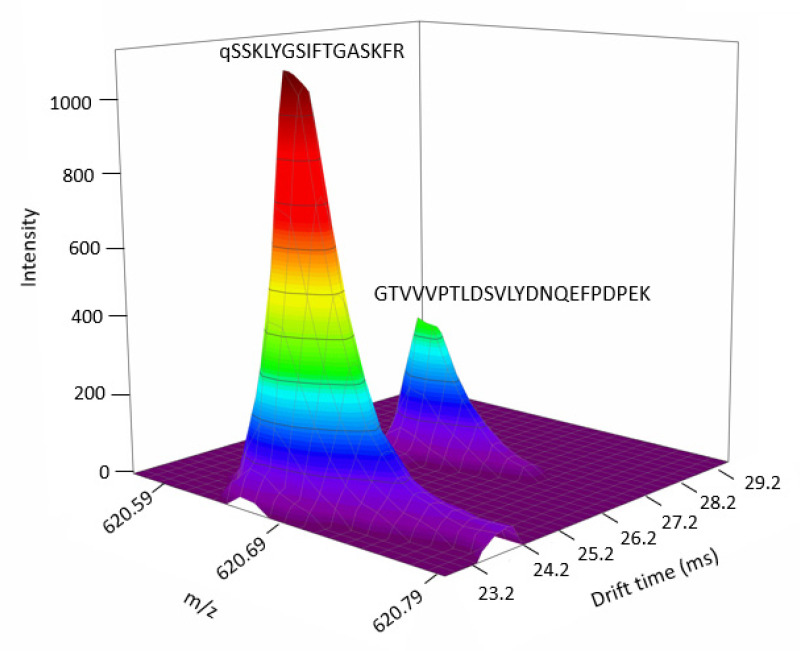
Separation of two isobaric peptides by the additional IM dimension. (Peptide 1 sequence: qSSKLYGSIFTGASKFR; *m*/*z*: 620.6703; RT: 89.54 min; DT: 23.57 ms.; Peptide 2 sequence: GTVVVPTLDSVLYDNQEFPDPEK; *m*/*z*: 620.6704; RT: 89.53 min; DT: 26.69 ms.

In this study, the use of DTIMS provided a third separation dimension that could allow the separation of the coeluting isobaric peptides. This ability was evaluated by analyzing a HeLa protein digest standard. The peptides were considered as coeluting and isobaric if their difference in retention time was not more than 0.25 min and their difference in *m*/*z* ratio was not more than 20 ppm. From analyzing the HeLa cells digest, a list of 3578 peptides was obtained. Among them, 41 pairs of coeluting isobaric peptides were identified. Thanks to the IM dimension, 24 pairs of coeluting isobaric peptides were separated among the 41 pairs (see Table S1, Supplementary Materials). Figure 6 represents an example of the separation of two coeluting isobaric peptides in the IM dimension. These results demonstrate the interest of DTIMS coupled to LC–MS to separate these closely related species and to be able to determine their concentration. This proteomics workflow will be applied to patient sample analysis in the near future. 

### 2.5. Interest of Multiplexed DIA in Proteomics

**Figure 7:**
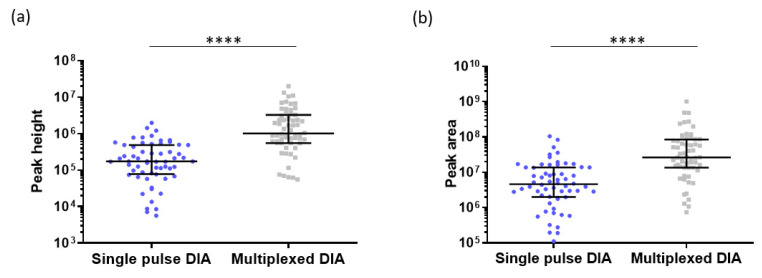
(**a**) Peak height of 60 representative peptides in single pulse DIA and in multiplexed DIA without CE; (**b**) Peak area of 60 representative peptides in single pulse DIA and in multiplexed DIA without CE. Analytical column: µPAC 50 cm; Sample: 500 ng HeLa Protein Digest Standard, Column temperature: 25 °C. Black lines represent the median with interquartile range. **** (*p*-value < 0.0001) (Mann–Whitney test).

Multiplexed DTIMS is another type of acquisition mode that was recently introduced. In multiplexed DTIMS, several packets of ions enter into the drift tube during each IM cycle [39]. This means that several packets of ions are present at the same time in the drift tube, which is different to single pulse DIA. The principle of single pulse DIA and multiplexed DIA are presented in Figure S3, Supplementary Materials. These small packets of ions are released in the drift tube according to a predetermined encoded sequence [39]. The duty cycle, which is the ratio between the trapping time and the time used for measuring, is increased in the multiplexed DTIMS compared to single pulse [39,40]. The multiplexed DTIMS can be used without any collision energy (CE) or with a fixed CE. If no CE is applied, only MS spectra are obtained and the peptide identification requires a peptide library. By the application of a fixed CE, MS and MS/MS spectra are obtained and the peptide identification does not require any previously developed library. In this study, the second option was chosen to compare the multiplexed-DIA and single-pulse DIA in terms of sensitivity. For this purpose, the peak height and peak area of 60 peptides, identified with single pulse DTIMS and with multiplexed DTIMS, were compared (Figure 7). As a result, the peak height was increased by a factor 5.8, using multiplexed DTIMS compared to single pulse, while the peak area was increased by a factor 5.7. Therefore, the multiplexed DTIMS can be an interesting approach to identify low concentrations of peptides and proteins. However, in the context of untargeted proteomics, one major issue with the multiplexed DTIMS is that the applied CE is fixed. 

The multiplexed DTIMS can therefore be an interesting and easy to use acquisition mode for samples which are weakly concentrated and for which it is not required to adapt the CE to each ion.

## 3. Materials and Methods

### 3.1. Chemicals and Reagents

Acetonitrile (ACN) and water (H_2_O) of LC–MS grade and formic (FA) acid and trifluoroacetic acid (TFA) of ULC–MS grade were obtained from Biosolve (Valkenswaard, The Netherlands). The difluoroacetic acid (DFA) of MS grade was purchased from Waters (Dublin, Ireland). The Pierce™ HeLa Protein standard digest was obtained from Thermo Fisher Scientific (Waltham, MA, USA). 

### 3.2. Nanofluidic Liquid Chromatography

Nanofluidic chromatographic analyses were performed on a 1200 series Agilent nano LC system. This system includes a capillary pump, a nanoflow pump, a column compartment, an autosampler and a HPLC–chip cube interface (Agilent Technologies, Waldbronn, Germany). MS calibration and Diagnosis Chip (Agilent Technologies) was used as the spray emitter. The analytes were ionized in a HPLC-chip source. The design of the analytical platform used in this study is represented in Figure S1, Supplementary Materials.

Mobile phase A and B of both the capillary and nanoflow pump contained H_2_O with 0.1% formic acid and ACN/H_2_O (90:10 *v*/*v*) with 0.1% FA, respectively. The flow rate of the capillary pump and the nanoflow pump were set at 10 µL/min and 0.3 µL/min, respectively. The peptides were separated in a 90 min gradient on the 50 cm µPAC column and in a 240 min gradient on the 2 m µPAC column. The gradient ranged from 8% to 38% of mobile phase B. The samples were kept at 4 °C in the autosampler before analysis. 

### 3.3. Mass Spectrometry Analysis

The experiments were achieved on a 6560 Ion mobility Q-TOF (Agilent Technologies, Waldbronn, Germany). A calibration chip (Agilent Technologies) was connected to the end of the µPAC column, thanks to the HPLC–chip cube interface (Agilent Technologies). The performances of this spray emitter were compared to the one obtained with a silica needle (PicoTip^TM^ emitter Silica Tip^TM^, FS 360-50-8-D-20, New Objective, Littleton, MA, USA). The analyses were conducted in positive electrospray ionization (ESI) mode. The gas flow was fixed at 4 L/min and gas temperature at 300 °C. The capillary voltage was set at 1900 V. Reference masses 121.0509 and 922.0098 *m*/*z* were continuously infused, thanks to an absorbent pad present on the HPLC-chip source. 

For DDA, the precursors were selected for fragmentation according to the following parameters: 3000 counts intensity threshold; 0.001% relative threshold; peptides; isolation width of four amu and max five precursors per cycle as the isotope model. The precursors were sorted by abundance only. CE was applied according to the charge state of each ion. For the ions with a charge state of one or two, the slope was set at 3.1 and offset at 1 while for ions with a charge state of three or more, the slope was set at 3.6 and offset at −4.8. 

For single pulse DIA, the drift tube was filled with nitrogen. The following parameters were applied: max drift time: 40 ms; drift tube entrance: 1574 V; trap fill time 38 000 µs; trap release time: 100 µs frame rate: 1.7 frame/s and IM transient rate: 15 transients/frame. MS and MS/MS spectra were acquired. For the MS/MS analysis, CE was adapted, according to the drift time. The linear ramp of CE was as follows: 0 ms → 8 V, 16 ms → 8 V, 40 ms → 65 V. 

For the four-bit multiplexed DIA, the drift tube entrance voltage was set at 1200 V, max drift time at 60 msec, trap fill time at 3900 µs, trap release time at 250 µs, frame rate at 1.1 frame/s and IM transient rate at 15 IM transients/frame. The acquisitions were made using a fixed CE of 31 V. 

### 3.4. Data Treatment

For the DDA analyses, raw data files were processed using Spectrum Mill software (Agilent Technologies). The peptides were identified by searching in the *Homo sapiens* database (SwissProt) using the following parameters: carbamidomethylation of cysteines as fixed modifications; acetylation of the N-terminal amino acid; oxidation of methionines; addition of a pyroglutamic acid and deamidation as the variable modifications; 25 % minimum matched peak intensity; 20 ppm precursor mass tolerance; 50 ppm product mass tolerance; trypsin digestion enzyme with a maximum of two missed cleavages. The reversed database scores calculation and dynamic peak thresholding were used. After peptide and protein identification, the annotations were validated using the auto threshold discriminant method with a global FDR < 1%. Only the peptides having a score higher than five and a scored peak intensity (SPI) higher than 60% were kept for data analysis. 

For single pulse DIA analyses, raw datafiles were first reprocessed in the IM-Reprocessor software (Agilent Technologies) for recalibration of the masses, using the following reference masses: 121.0509 and 922.0098 *m*/*z*. After reprocessing, the data were smoothed on the LC and IM dimensions using the PNNL PreProcessor (beta 2020.11.24) (Pacific Northwest National Laboratory, Richland, WA, USA) with three points smoothing for the LC dimension and five points smoothing for the IM dimension. After single-field CCS calibration, the smoothed datafiles were imported into the IM-Browser 10.0 software to apply a 4D-ion mobility feature extraction algorithm (4D-IMFE) to identify the ions with a peptide profile (charge state between two and seven). In this algorithm, the intensity threshold was set at 50 counts. After this feature-finding process, the precursor ions and product ions were aligned, based on their retention time (±10 s) and their drift time (±0.5 ms), with maximum 25 peaks per MS/MS spectrum. The output files (.pkl files) were then processed in the Spectrum Mill software for peptide and protein identifications (same parameters as for DDA). 

For the multiplexed DIA analyses, the raw datafiles were interpolated, demultiplexed and smoothed using the PNNL PreProcessor (beta 2020.11.24) (Pacific Northwest National Laboratory, Richland, WA, USA). Drift bin interpolation was applied so that one drift bin became three drift bins. This interpolation step enhances the effective sampling rate of the IM dimension. The data were smoothed, using three points smoothing for the LC dimension and five points smoothing for the IM dimension. The features were then detected in the IM-Browser 10.0 software, using the same parameters as for single-pulse DIA. The features list was exported and used for high resolution demultiplexing, using the new HRdm 2.0. software developed by Agilent Technologies in order to increase considerably the resolution in the IM dimension. For this high-resolution demultiplexing step, HR processing level used was high, *m*/*z* width multiplier was set at 5, sat threshold multiplier at 0.5 and IF multiplier at 1. Check saturation was enabled. After this step of resolution improvement, mass recalibration (IM Reprocessor software) as well as single field CCS calibration (IM-Browser software) was applied to the output files. The features were then detected on the HRdm output file in the IM-Browser software followed by precursor and product ions alignment, both using the same parameters as for single pulse DIA. Finally, the peptides were identified, using the Spectrum Mill software with the same parameters as previously described for the DDA and single pulse DIA. The whole data treatment process is summarized in Figure S2, Supplementary Materials. 

### 3.5. Comparison of Two Trapping Columns 

A µPAC trapping column (Pharmafluidics, Ghent, Belgium) or a Zorbax 300SB-C18, 5 × 0.3 mm, 5 µm, enrichment column (Agilent Technologies) was connected to a 2 m µPAC column. Pierce™ HeLa Protein Digest Standard was resuspended in H_2_O containing 0.1% FA at a concentration of 0.125 µg/µL and the injection volume was set at 8 µL. The analyses were achieved in DDA mode.

### 3.6. Influence of the Acidic Modifier in Sample Loading Mobile Phase and Sample Resuspending Solvent

Pierce™ HeLa Protein Digest Standard was resuspended in H_2_O containing 0.1% FA, DFA or TFA at a concentration of 0.125 µg/µL and the injection volume was set at 8 µL. The mobile phase A of the capillary pump contained H_2_O with 0.1% FA, DFA or TFA and the mobile phase B contained ACN/H_2_O (90:10 *v*/*v*) with 0.1% FA, DFA or TFA according to the acid used in the resuspending solvent. The peptides were trapped on a µPAC trapping column and analyzed on a 2 m µPAC column. The analyses were achieved in DDA mode.

### 3.7. Optimization of Sample Resuspending Solvent

Pierce™ HeLa Protein Digest Standard was resuspended in four different resuspending solvents (RS). RS 1 contained H_2_O with 0.1% TFA. RS 2 consisted of H_2_O/ACN (95:5 *v*/*v*) with 0.1% TFA. RS 3 contained H_2_O/ACN (95:5 *v*/*v*) with 0.1% FA and RS 4 H_2_O/ACN (95:5 *v*/*v*) with 0.1% FA and 0.05% TFA. 1 µg of peptides were trapped on a µPAC trapping column and analyzed on a 2 m µPAC column. The analyses were achieved in DDA mode.

### 3.8. Comparison of Two µPAC Column Lengths and Impact of the Temperature 

500 ng of Pierce™ HeLa Protein Digest Standard were resuspended in H_2_O/ACN (95:5 *v*/*v*) with 0.1% FA and injected on a 50 cm µPAC column and a 2 m µPAC column. The analyses were achieved in DDA and DIA modes. 

## 4. Conclusions

In this study, µPAC columns were coupled to DTIMS and QTOF–MS to identify as many peptides and proteins as possible in a complex protein digest standard. A chip was used as an emitter to stabilize the spray. Several chromatographic parameters that have an impact on the number of identifications were optimized. The C18 µPAC trapping column was chosen over C18 Zorbax trapping column, since it provided more identifications. Besides, the sample-loading mobile phase and sample-resuspending solvent were investigated, as well as the impact of the µPAC column length. For clinical studies conducted on large cohorts of patients, a 50 cm long µPAC column seems to be a good compromise between a high number of identifications and a reasonable analysis time. Furthermore, the complementarity of DDA and single pulse DIA modes was demonstrated. Adding the IM dimension to the nano LC–MS increased considerably the number of peptides and proteins identifications. Moreover, DTIMS provides a third dimension of separation and can be very interesting to separate coeluting isobaric peptides to better understand several pathologies. Regarding the multiplexed DTIMS, the sensitivity was considerably increased and constitutes, therefore, an innovative and promising approach to analyze proteins present in low concentrated samples and/or in very low sample volume. 

In conclusion, the developed nano LC–DTIMS platform could be applied to complex patient samples to decipher the molecular pathways involved in several diseases, or to answer complex biological questions that require high sensitivity.

## Data Availability

The data presented in this study are available on request from the corresponding author. The data are not publicly available due to their size.

## Supplementary Materials

The following supporting information can be downloaded at: https://www.mdpi.com/article/10.3390/ijms23147497/s1. Figure S1: Design of the analytical platform used in this study; Figure S2: Data treatment process; Figure S3: Principle of single pulse DIA and multiplexed DIA; Table S1: Pairs of non-isomeric coeluting isobaric peptides that could be separated thanks to the ion-mobility dimension; Table S2: List of the 60 peptides used to compare single pulse and multiplexed DIA.
