# Supplementary material for: Potential of Single Pulse and Multiplexed Drift-Tube Ion Mobility Spectrometry Coupled to Micropillar Array Column for Proteomics Studies
Source: Int J Mol Sci. 2022 Jul 6;23(14):7497. doi: 10.3390/ijms23147497 (PMC9319919; doi:10.3390/ijms23147497)
Supplement: Supplementary file 1 [file ijms-23-07497-s001.zip › ijms-1781501-supplementary.pdf]

Supplementary Materials

Figure S1. (a) Design of the analytical platform used in this study. (b) Connection between the µPAC and the calibration chip by the HPLC Chip-cube.

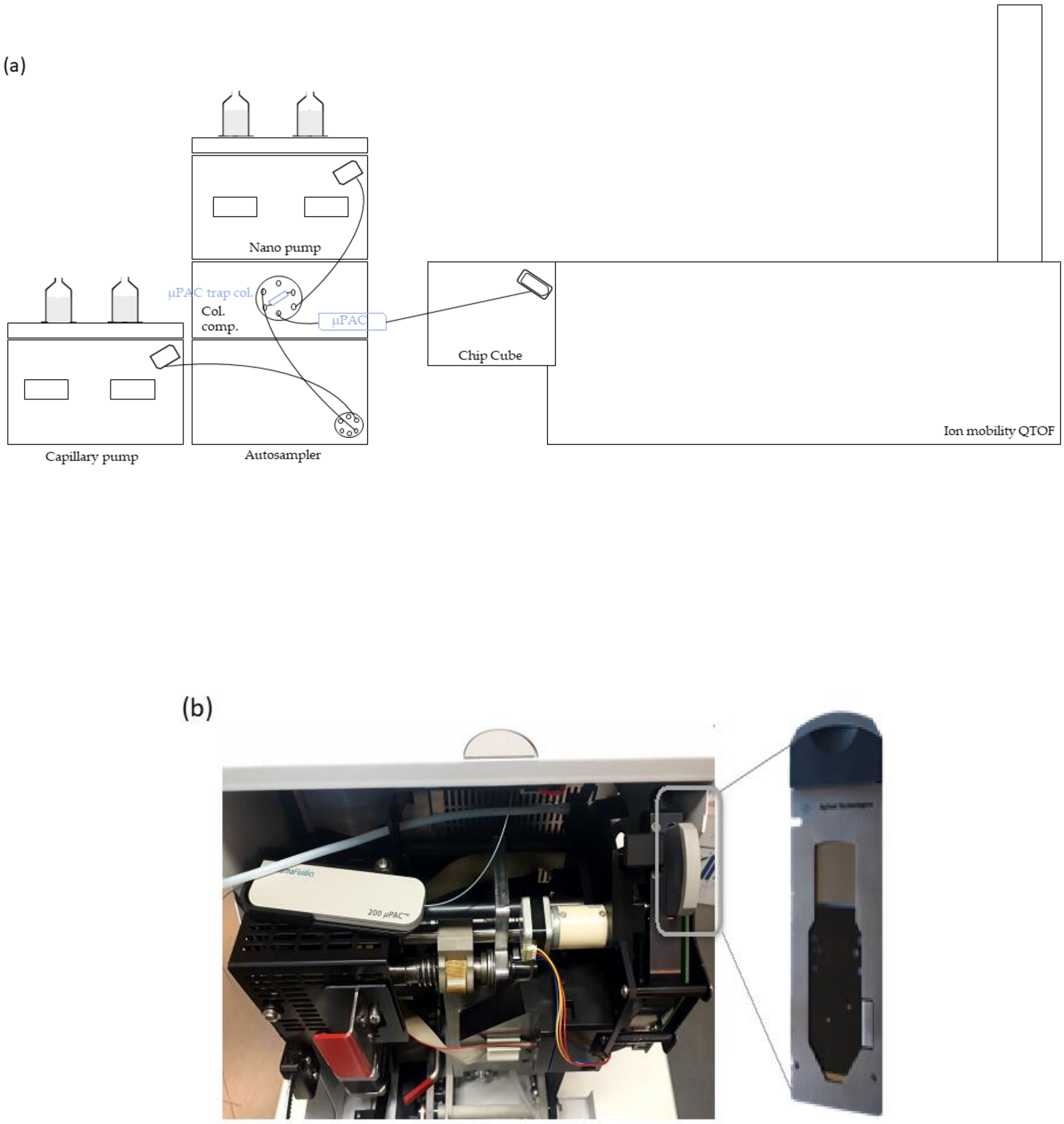

Figure S2. Data treatment process

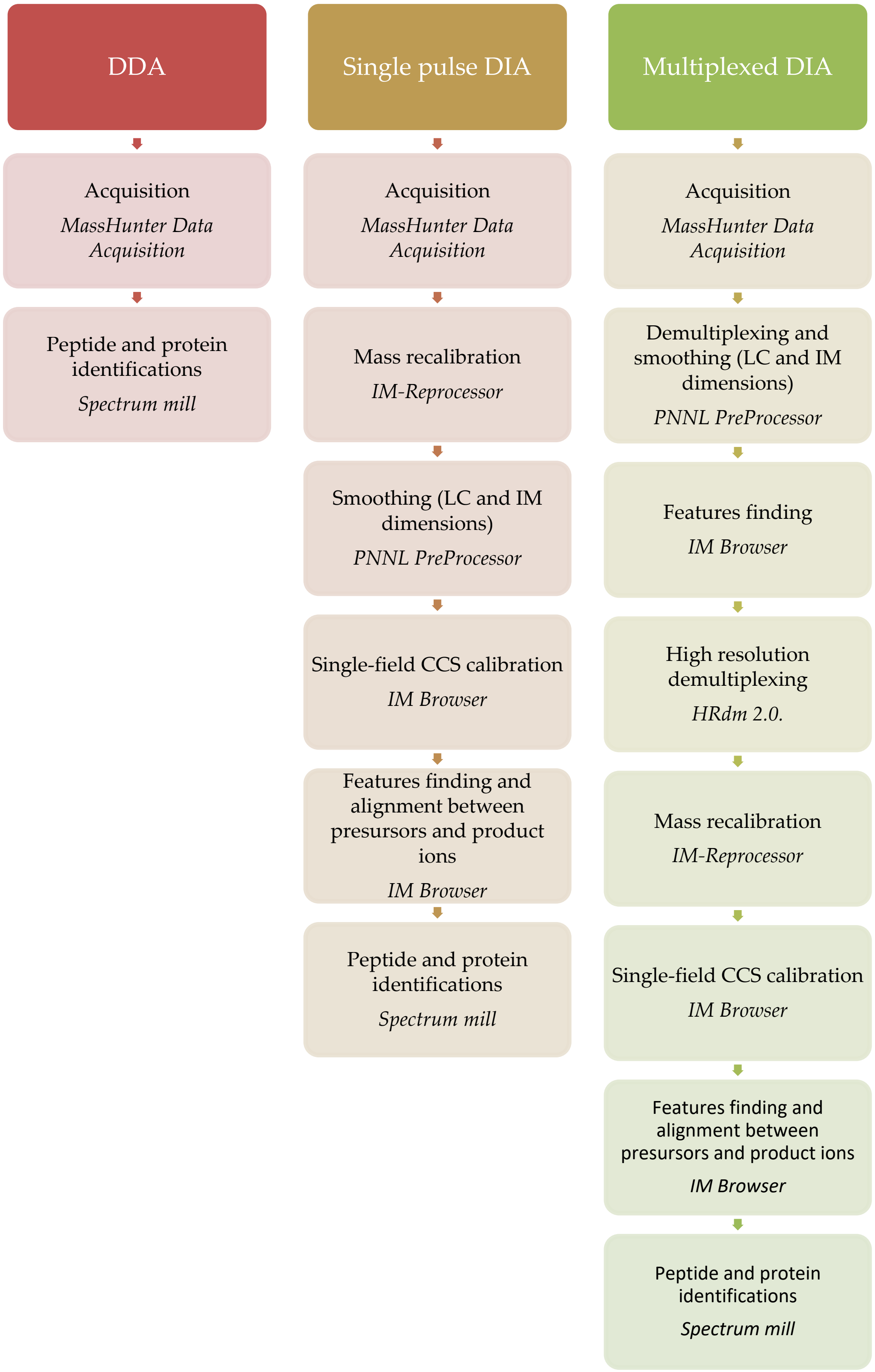

Figure S3. Principle of single pulse DIA and multiplexed DIA. In single pulse DIA: one packet of ions into the drift tube during each IM cycle. In multiplexed DIA: several packets of ions introduced into the drift tube during each IM cycle. Green, red and yellow dots represent different types of ions.

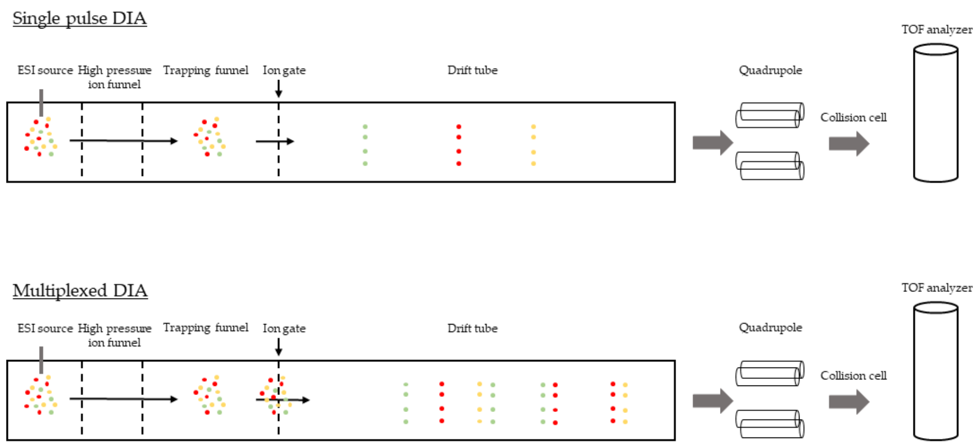

Table S1. Pairs of non-isomeric coeluting isobaric peptides that could be separated thanks to the ion-mobility dimension. RT= retention time; DT= drift-time; CCS= collision-cross section. Cysteines are carbamidomethylated (fixed modification).

Sequence	m/z	RT (min)	DT (ms)	CCS	Charge	Variable Modifications
**VLYGLFCKHTQK**	498.5978	61.10	20.68	455.49	3	
**YGFTHLSTGELLR**	498.5987	61.11	22.58	498.36	3	
**YDLYEKTLKYnSDLLnSnR**	588.7894	42.80	23.04	675.94	4	Deamidation
**SFSLGDIYFK**	588.7891	43.02	20.89	307.59	2	
**FPIYHLLLSGNLLNR**	590.6625	74.43	23.01	507.29	3	
**RAnPHFKSFPVLEAR**	590.6626	74.45	24.25	535.31	3	Deamidation
**IGSEIGCYQGFAFAWK**	611.9697	66.55	27.20	601.58	3	
**TYFPHFDLSHGSAQVK**	611.9713	66.57	21.93	482.85	3	
**GTVVVPTLDSVLYDNQEFPDPEK**	620.6704	89.53	26.69	590.13	3	
**qSSKLYGSIFTGASKFR**	620.6703	89.54	23.57	519.90	3	Pyroglutamic acid
**VHTGEKPYECFECPK**	627.6176	79.36	25.47	562.64	3	
**DTYSPDEHFWVTLnR**	627.6186	79.46	23.00	506.90	3	Deamidation
**QYGGFDnRGLEPGHR**	634.3355	63.24	22.74	501.05	3	
**NLYGAKSLELPFQGAHR**	634.3360	63.26	21.88	481.58	3	
**GYWTQLSmLIPGPGFSR**	642.6581	74.91	22.63	498.52	3	Oxidized methionine
**FSFHTNVLEDnIGYLR**	642.6559	74.91	24.26	535.20	3	Deamidation
**ELISNSSDALDK**	646.3233	43.34	25.93	383.19	2	
**DKYPLSEnHNnnTFLK**	646.3150	43.59	24.49	540.35	3	Deamidation
**IAEIFHLNSVSGEVSILK**	652.6960	64.28	20.96	460.80	3	
**VAPEEHPTLLTEAPLNPK**	652.6963	64.32	25.02	552.24	3	
**ELTVYGnPICRKMLHR**	663.3478	67.52	24.67	544.21	3	Deamidation
**ILGPmFISQVYAHWGPR**	663.3466	67.57	23.41	515.85	3	Oxidized methionine
**HPNTLLRRGAFLSYSQK**	663.3665	89.10	23.95	528.19	3	
**IASLDAAnARLMSALTQLK**	663.3663	89.11	26.09	576.30	3	Deamidation
**YFHRIDnFLK**	677.3394	53.16	20.53	301.69	2	Deamidation
**DIISDTSGDFRK**	677.3366	53.16	26.76	395.51	2	
**GRnnmFHTLLmFLYIIK**	715.6863	89.34	23.52	518.18	3	Deamidation Oxidized methionine
**qISDVERDVQNFPAATDEK**	715.6890	89.37	26.39	582.78	3	Pyroglutamic acid
**QmGYPVLNVnGVKnITQKR**	726.3880	83.45	25.25	557.08	3	Deamidation Oxidized methionine
**EIIVFVESFIYKHPQEAK**	726.3866	83.48	27.71	612.49	3	
**YTLLNFVPRnLFEQFHR**	732.3954	78.58	25.08	553.22	3	Deamidation
**HQLVLDHHVKGHGGTRLYK**	732.3953	78.60	26.89	593.93	3	
**TMNLNPmILTNILSSPYFK**	738.3766	82.04	24.79	546.74	3	Oxidized methionine
**VIISAPSADAPMFVMGVNHEK**	738.3762	82.04	27.43	606.06	3	
**QHGTYRQIFHPEQLITGKEDAAnnYAWGHYTIGK**	789.9824	84.11	27.46	1008.36	5	Deamidation
**TSEIEPKnSPEDLGLSLTGDSCKLmLSTSEYSQSPK**	789.9829	84.11	29.25	1075.21	5	Deamidation Oxidized methionine
**QYEHVKRDLNPEDFWEIIGELGDGAFGK**	816.3870	121.64	26.98	792.84	4	
**QYEHVKRDLNPEDFWEIIGELGDGAFGK**	816.3861	121.64	24.92	731.28	4	
**GSAGPCCTPTKMSPInmLYFNGKEQIIYGK**	845.6613	80.46	27.00	793.41	4	Deamidation Oxidized methionine

**SYVEGFGDPMKEFWLGLDKLHNLTT GTPAR**	845.6621	80.51	24.40	715.69	4	
**HSTINIVDIYPMMCHILGLKPHPnNG TFGHTK**	911.6913	96.27	29.47	867.06	4	
**DLSKTIIIDnSPQAFAYQLSnGIPIESW FMDK**	911.6933	96.30	26.08	765.71	4	
**SLDLFNCEVTNLNDYR**	986.9602	91.31	31.73	468.85	2	
**FYLTPPQVnSILK**	986.9578	91.32	28.32	417.63	2	Deamidation
**IIPTLEEGLQLPSPTATSQLPLESDAV ECLNYQHYK**	1014.5142	112.62	33.14	976.56	4	
**WIISDGPVDALWIEnmNTVLDDnKm LCLANSERIK**	1014.5148	112.63	28.87	848.75	4	Deamidation Oxidized methionine
**INFTLRVHDVLFGDVWLCSGQSnmQ mTVLQIFnATR**	1062.0015	112.74	27.13	796.54	4	Deamidation Oxidized methionine
**EAVEKEFEPLLnWmKDK**	1062.0024	112.75	28.70	423.21	2	Deamidation Oxidized methionine

Table S2. List of the 60 peptides used to compare single pulse and multiplexed DIA. Cysteines are carbamidomethylated (fixed modification).

**Sequence**	**m/z**	**RT (min)**	**Charge**
IGAEVYHNLK	381.8773	39.10	3
FSMPGFK	407.2012	63.60	2
VIFGLFGK	440.7693	85.10	2
GLFIIDDK	460.7536	68.66	2
LPLQDVYK	488.2766	52.68	2
DLPLLLFR	493.8033	104.13	2
VFLENVIR	495.2872	68.77	2
SLTNDWEDHLAVK	509.9208	65.40	3
VSFELFADK	528.2734	76.77	2
MFLSFPTTK	536.2815	76.23	2
LHFFMPGFAPLTSR	540.9504	98.57	3
EIIDLVLDR	543.3117	83.04	3
LSELEAALQR	565.3106	59.47	2
ISGLIYEETR	590.8131	53.02	2
VIGSGCNLDSAR	624.7977	35.88	2
AGKPVICATQMLESMIK	626.3317	101.98	3
DFMIQGGDFTR	643.7932	67.89	2
TVTAMDVVYALK	655.8593	87.45	2
ITLPVDFVTADK	659.8632	83.44	2
SLDMDSIIAEVK	660.8454	89.16	2
GFAFVQYVNER	665.3420	76.28	2
YALYDATYETK	669.3175	52.55	2
FLSQPFQVAEVFTGHMGK	675.0104	103.79	3
NTGIICTIGPASR	680.3583	52.53	2
IQTQPGYANTLR	681.3673	41.56	2
SEPIPESNDGPVK	684.8369	37.53	2
LLTSFLPAQLLR	686.4249	108.15	2

ALMLQGVDLLADAVAVTMGPK	705.0622	123.47	3
DICNDVLSLLEK	709.8705	112,63	2
ILTFDQLALDSPK	730.9014	85.22	2
LICCDILDVLDK	738.8755	99.55	2
SETAPAAPAAAPPAEK	760.8797	37.99	2
LVQSPNSYFMDVK	764.3793	68.55	2
VPTANVSVVDLTCR	765.8994	63.81	2
ADQLTEEQIAEFK	782.3913	76.06	2
IQPSGGTNINEALLR	791.9331	53.11	2
SETAPAETATPAPVEK	820.9050	39.32	2
DPVQEAWAEDVDLR	821.8834	77.42	2
GTDIMYTGTLDCWR	844.8781	77.94	2
AAGVNVEPFWPGLFAK	851.9423	107.65	2
GCITIIGGGDTATCCAK	877.8944	51.35	2
DCDLQEDACYNCGR	888.3182	39.92	2
SYELPDGQVITIGNER	895.9506	78.95	2
LEAELGNMQGLVEDFK	896.9475	96.86	2
EKPYFPIPEEYTFIQNVPLEDR	908.8025	102.85	3
MTDQEAIQDLWQWR	910.4303	104.58	2
SNMDNMFESYINNLR	924.3993	103,63	2
YVASYLLAALGGNSSPSAK	934.9900	96.78	2
TDITYPAGFMDVISIDK	943.4650	102.42	2
MEDSMDMDMSPLRPQNYLFGCELK	983.7589	111.62	3
SLDLFNCEVTNLNDYR	986.9519	91.19	2
AAFDDAIAELDTLSEESYK	1044.4826	110.27	2
MVNPTVFFDIAVDGEPLGR	1060.033	122.23	2
EMEENFAVEAANYQDTIGR	1093.9890	76.56	2
DLYANTVLSGGTTMYPGIADR	1108.0494	83,69	2
GVVPLAGTNGETTTQGLDGLSER	1136.5656	69.05	2
DGELPVEDDIDLSDVELDDLGK	1201.0574	99.46	2
GFEVVYMTEPIDEYCVQQLK	1224.5667	93.09	2
LNSNTQVVLLSATMPSDVLEVTK	1230.1526	100.82	2
TALLDAAGVASLLTTAEVVVTEIPK	1241.6861	124.68	2
